# S40. MEDIAL SEPTUM ACTIVATION PRODUCES OPPOSITE EFFECTS ON DOPAMINE NEURON ACTIVITY IN THE VENTRAL TEGMENTAL AREA AND SUBSTANTIA NIGRA PARS COMPACTA IN MAM VERSUS NORMAL RATS

**DOI:** 10.1093/schbul/sby018.827

**Published:** 2018-04-01

**Authors:** David Bortz, Anthony Grace

**Affiliations:** University of Pittsburgh

## Abstract

**Background:**

Disruptions in dopamine (DA) signaling are central to the pathophysiology of several major psychiatric disorders, including schizophrenia. Thus, discovery of novel therapeutic approaches that normalize DA signaling is a major focus of research. One pathway that is critical for DA regulation originates from the ventral subiculum (vSub) of the hippocampus and controls ventral tegmental area (VTA) DA neuron activity. A potent regulator of hippocampal function is the medial septum (MS); a sub-region of the basal forebrain that widely innervates the hippocampus, including the vSub, via cholinergic, GABAergic, and glutamatergic projections, drives hippocampal theta rhythms, and affects goal-directed learning and memory. Despite this, it has never been determined if the MS is an afferent regulator of the midbrain DA system, and therefore may be a novel therapeutic treatment target for DA-related disorders.

**Methods:**

Effects of MS activation (NMDA, 0.75 µg/ 0.2 µL) were examined in intact and methylazoxymethanol acetate- treated (MAM) male Sprague-Dawley rats using anesthetized single unit DA recordings in the VTA and substantia nigra pars compacta (SNc) and locomotor behavior in an open field following systemic amphetamine (0.75 mg/kg).

**Results:**

MS activation produced a prolonged 71% increase in the number of spontaneously active DA cells in the VTA, and an opposing 40% decrease in the number of active DA cells in the SNc, compared to vehicle infusions. These effects were mediated by the vSub and ventral pallidum as local infusion of TTX (1 µM /0.5 µL) and bicuculline (1 ng/ 0.5 µL), respectively, reversed DA population activity changes in both regions. MS activation also decreased the locomotor response to amphetamine (49% reduction in distance traveled during peak ambulation compared to vehicle). MS activation-induced changes in both DA population activity and amphetamine-induced hyperlocomotion were selectively mediated by different neurotransmitter populations from MS to vSub as infusion of scopolamine (8 µg/1.0 µL) into the vSub selectively prevented DA population activity changes in the VTA. In contrast, infusion of bicuculline (12.5 ng/0.5 µL) selectively prevented DA population activity changes in the SNc and the decrease in amphetamine-induced hyperlocomotion. In MAM rats, MS activation produced opposite effects on DA population activity versus controls as it decreased VTA DA activity by 51% and increased SNc DA activity by 47%. This was accompanied by a similar reversal in amphetamine-induced hyperlocomotion, with MS activation increasing locomotion in MAM animals (113% increase in distance traveled during peak ambulation compared to control animals). The reversal in behavioral output is likely due to disrupted GABAergic projections from MS to vSub as bicuculline infusion into vSub prevented the increase in locomotor behavior.

**Discussion:**

These data indicate that the MS differentially regulates both VTA and SNc DA neuron activity and behavioral output, but via distinct pathways, and that this regulation is disrupted in a well-validated animal model of schizophrenia. Therefore, this suggests that in normal animals the MS might activate the VTA to increase information processing while delaying action via SNc inhibition. In contrast, the inhibition of VTA and activation of SNc in MAM rats might promote a rapid response without adequate processing of information.

